# Beyond the Unitary: Direct, Moderated, and Mediated Associations of Mindfulness Facets with Mental Health Literacy and Treatment-Seeking Attitudes

**DOI:** 10.3390/healthcare13101201

**Published:** 2025-05-20

**Authors:** Matea Gerbeza, Kelsy Dąbek, Katelyn Lockinger, Isabelle M. Wilkens, Mia Loarca-Rodriguez, Katimah Grogan, Shadi Beshai

**Affiliations:** 1 Department of Psychology, University of Regina, Regina, SK S4S 0A2, Canada; kmd695@uregina.ca (K.D.); ksl766@uregina.ca (K.L.); sbeshai@kennesaw.edu (S.B.); 2 Department of Psychological Science, Kennesaw State University, Kennesaw, GA 30144, USA; iwilkens@students.kennesaw.edu (I.M.W.); kgroga10@students.kennesaw.edu (K.G.)

**Keywords:** dispositional mindfulness, mental health literacy, treatment-seeking attitudes, self-efficacy, mindfulness facets, five-facet mindfulness questionnaire (FFMQ)

## Abstract

Background and Objectives: Psychological disorders are prevalent and distressing. Early treatment initiation can prevent adverse outcomes and reduce healthcare system impacts. Improving mental health literacy (MHL)—one’s knowledge regarding psychological disorders—and treatment-seeking attitudes (TSAs) is key in early treatment initiation. Examining the facets of dispositional mindfulness—the capacity to pay attention to present-moment experiences with acceptance—may offer more granular insights into understanding MHL and TSAs. This study examined (a) associations between mindfulness facets and MHL and TSAs, (b) facets’ prediction of MHL and TSAs beyond demographics, (c) moderation of the MHL–TSA relationship by mindfulness facets, and (d) mediation of mindfulness–TSA relationships via general self-efficacy (GSE). Methods: A community sample of 299 adults was recruited online (TurkPrime) and completed demographic questions and self-report measures: Five-Facet Mindfulness Questionnaire-15, Mental Health Literacy Scale, Mental Help-Seeking Attitudes Scale, and General Self-Efficacy Scale. Results: Describe, Non-Judgment, and Act with Awareness were modestly associated with MHL; all five facets correlated with TSAs. Hierarchical regressions controlling for demographics showed that Describe and Non-Reactivity predicted MHL, while Act with Awareness uniquely predicted TSAs. Non-Reactivity moderated the MHL–TSA relationship, with higher Non-Reactivity amplifying the relationship. GSE fully mediated relationships between Observe and Non-Judgment with TSAs, suggesting self-efficacy is a key mechanism of these facets. Conclusions: Interventions cultivating Non-Reactivity, Describe, and Act with Awareness may improve the translation of mental health knowledge into treatment-seeking behaviors. Future research should explore how mindfulness facets independently and interactively foster early intervention and treatment engagement.

## 1. Introduction

Psychological disorders are prevalent, distressing, and significantly disruptive to daily functioning [1]. Unfortunately, symptoms of psychological disorders are not isolated; those who experience elevated symptoms are also more susceptible to developing physical health conditions, such as cardiovascular conditions, cancers, and other chronic disorders [2,3]. Negative impacts of psychological disorders are widespread, leading to reduced life expectancy and increased risk for suicide [4]. Unaddressed or delayed treatment of psychological disorder symptoms has a substantial negative impact, and has accrued costs for individuals, societies, and healthcare systems [5]. Early intervention has been shown to reduce the burden and impact of psychological disorders on individuals and society [6].

Despite a growing effort in supporting mental health service initiatives led by institutions, there remain significant barriers to accessing services or obtaining accurate information [7]. Beyond systemic barriers, interpersonal factors such as attitudes, intentions, and behaviors related to psychological disorders or treatment-seeking are critical. Negative attitudes towards or a lack of knowledge about psychological disorders may create reluctance towards treatment-seeking [7]. Promotion and cultivation of mental health literacy (MHL) is key to preventing psychological distress. MHL is a broad, multifaceted concept. A key pillar of MHL is one’s knowledge, beliefs, attitudes, and abilities to recognize specified factors surrounding mental health [8]. MHL includes the knowledge and beliefs surrounding risk factors and causes; self-help interventions; availability of professional help; knowledge of how to seek information on psychological disorders; attitudes facilitating recognition and appropriate help-seeking; and abilities in recognizing specific disorders or varying states of psychological distress [8]. Therefore, high levels of MHL have the potential to decrease psychological distress by promoting better detection of symptoms and early treatment-seeking behaviors through enhancing knowledge among laypersons.

Many with psychological distress do not receive or seek help, and when they do, they do not seek professional services [9,10]. Attitudes and intentions surrounding treatment-seeking are pivotal in determining whether individuals access mental health services. Although various factors (i.e., affordability, accessibility) can influence this decision, MHL emerges as a particularly consistent predictor. Researchers found that MHL was the second-strongest predictor of help-seeking intention and the strongest predictor of treatment-seeking attitudes (TSAs) [11].

According to the World Health Organization [4], improving MHL is key in preventing consequences surrounding psychological disorders. In a study of psychologically distressed older adults, Mackenzie et al. [12] found that individuals with lower MHL exhibited reduced treatment-seeking behaviors and increased self-stigmatization, potentially compounding psychological distress. By contrast, targeted MHL interventions were associated with fewer psychological disorder symptoms [12]. Early treatment-seeking has been associated with better long-term outcomes [13]. Furthermore, a systematic review by Magallón-Botaya et al. [14] revealed that MHL interventions significantly reduced symptoms of depression and anxiety. This lends further support to the notion that MHL can lead to improved mental health outcomes for individuals and healthcare systems [14].

Accordingly, MHL is robustly associated with an increased overall inclination to access mental health help. However, other factors, such as demographic characteristics, sociocultural context, and dispositional traits, may also play a role in how individuals acquire and apply MHL. Given that recognition and acknowledgment of psychological distress are critical aspects of MHL and treatment initiation, it stands to reason that personal qualities that enhance individuals’ awareness of their own cognitions and emotions, such as mindfulness, may be associated with MHL. Mindfulness is commonly defined as paying attention to internal and external present-moment experiences with an attitude of openness, acceptance, and curiosity [15,16]. Dispositional mindfulness (DM) is a naturally occurring trait characterized by an individual’s ability to regulate their attention to present-moment experiences with an attitude of acceptance [17,18]. Although various conceptualizations of mindfulness exist, empirical evidence has provided support for multifaceted models of DM [19,20,21]. One such model is the five-factor model of mindfulness, often measured by the Five-Facet Mindfulness Questionnaire (FFMQ) [19]. The FFMQ assesses five distinct but correlated facets: Observe (attending to thoughts, emotions, and sensations), Describe (labeling internal experiences), Act with Awareness (fully engaging in activities rather than being mindless or engaging in habitual responding), Non-Judgment (adopting a non-evaluative stance to experience), and Non-Reactivity (allowing thoughts and emotions to come and go without attachment) [19]. Given DM’s broad and multifaceted nature, examining it as a unitary construct in the context of important clinical correlates may prevent researchers from achieving granularity in understanding these relationships. This poor granularity may in turn provide little insight regarding optimal MHL intervention development or refinement.

**Figure 1:**
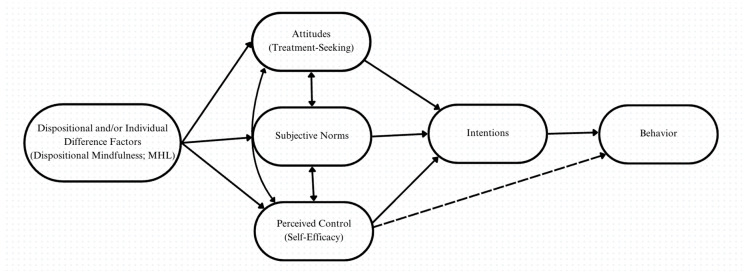
Theory of Planned Behavior framework for the present study.

Theoretically, DM may support MHL and TSAs through several mechanisms. First, by promoting non-judgmental awareness of present-moment experiences, DM may improve individuals’ recognition of psychological distress, an essential component of MHL [17]. Second, the beneficial effects of DM in relation to MHL and treatment-seeking may be explained using the Theory of Planned Behavior (TPB) [22]. The TPB posits that attitudes, norms, perceived behavioral control, and intentions interact to predict one’s behavior [22]. Within the framework of the TPB, DM or other trait factors may be distally associated, either directly or indirectly, with attitudes, subjective norms, and perceptions of control over mental health concerns. These associations are conceptualized to influence positive intentions toward treatment-seeking. This may ultimately lead to adaptive behavior among individuals facing psychological distress (Figure 1). There are several supportive lines of evidence for this potential relationship. Bowlin and Baer [23] found that DM was positively associated with perceptions of self-control and that it moderated the relationship between self-control and psychological well-being. Further, Short et al. [24] found that DM was associated with improved self-regulation and that self-regulation mediated the mindfulness–well-being relationship. Accordingly, DM and heightened MHL may be critical predictors of improved TSAs, which in turn are vital for taking practical actions toward seeking support for psychological symptoms.

In direct support of the TPB, self-efficacy is consistently associated with improved intentions to change health and practical health change behaviors [25]. Self-efficacy is defined as an individual’s belief in their ability to successfully perform tasks, achieve goals, and manage challenges [26]. High self-efficacy has been found to be correlated with better coping with chronic conditions (e.g., diabetes) and high rates of quality of life [27,28]. Self-efficacy is also correlated with a sense of internal control over psychological symptoms [29]. Moreover, there is evidence to suggest that DM may be associated with higher levels of self-efficacy. Chandna et al. [30] found that the Non-Reactivity, Act with Awareness, Observe, and Describe facets positively predict self-efficacy. The TPB [22] posits that dispositional factors (such as DM and its facets) may lead to stronger intentions or more favorable TSAs, with this effect potentially mediated by an individual’s general sense of self-efficacy and perceived control over health behaviors.

As mentioned, DM is not a unitary construct, and evidence suggests each of the facets has a differential relationship with symptoms of psychopathology and their correlates. For example, a meta-analysis by Carpenter et al. [31] found that Non-Judgment and Act with Awareness have the strongest negative relationships with symptoms, followed by Non-Reactivity. Observe did not show a systematic relationship with symptoms. Act with Awareness and Non-Judgment have also displayed the strongest negative relationships with emotional dysregulation [32]. Pertinently, Karl et al. [33] found that Describe, Act with Awareness, and Non-Judgment were more strongly negatively associated with alexithymia (difficulty in the recognition and description of emotions) compared to other facets of mindfulness. Taken together, the extant literature on the correlates of the facets, while scarce, shows a consistent pattern. Among non-meditating samples, Observe tends to display weaker relationships with symptoms but may have other interactive properties that make correlates of mindfulness (or the effects of other facets) more pronounced [19]. Describe appears to provide emotional clarity and hence may lead to improved emotional regulation [33]. Act with Awareness, which captures the attentional regulation aspects of mindfulness, is robustly associated with reduced symptoms [31]. Non-Judgment is more directly and strongly associated with reduced symptoms and therefore may be less interactive in its effects compared to other facets [31]. Finally, Non-Reactivity seems to have an instrumental role in helping patients be less reactive to their own distress. Each facet of DM may have unique relationships with MHL and TSAs. FFMQ facets related to enhanced awareness—Observe, Describe, and Act with Awareness—may relate more closely to MHL. By contrast, attitudinal components of DM, such as Non-Judgment and Non-Reactivity, may have greater predictive utility regarding TSAs. However, there is an absence of research examining the direct or indirect relationships of mindfulness facets with self-efficacy, MHL, and TSAs.

Research examining the association between DM and MHL is limited; however, existing research is suggestive. For instance, DM is positively associated with emotional intelligence, which reflects an individual’s ability to understand, address, and regulate their emotions [34]. The ability to direct one’s attention to and understand their emotions, including the contributing causes, may closely align with the knowledge and recognition components of MHL. Lo et al. [35] found that DM is positively correlated with MHL, demonstrating that the ability to describe and label one’s internal states, including psychopathological symptoms, may help individuals recognize that they are experiencing distress. When this awareness is intentional and non-judgmental, individuals may be more likely to address such symptoms. Consistent with this notion, Blignault et al. [36] found that participation in a mindfulness-based intervention led to improved MHL and treatment-seeking behaviors. These findings align with previous findings that high levels of DM are associated with various adaptive behaviors including healthy life skills (e.g., health literacy) [37], effective emotional regulation [38], and active coping strategies (e.g., self-help, problem-solving) [39].

Attitudinal barriers are among the most common and influential deterrents to treatment-seeking [40]. Accepting attitudes characteristic of individuals high in DM may facilitate positive attitudes toward treatment. For instance, DM has been identified as a moderator of the relationship between psychological distress and self-stigma, such that the positive association between distress and stigma was attenuated among individuals high in DM [41]. DM and its facets have been associated with reduced levels of stigma among schizophrenia patients [42]. Specifically, Tang et al. [42] found that all DM facets were associated with reduced perceptions of discrimination, Act with Awareness was the only facet associated with lower stigma coping (e.g., withdrawal), and Non-Judgment was uniquely associated with reduced stigma-related feelings (e.g., shame). Non-Judgment, Observe, Describe, and Non-Reactivity also displayed moderate positive correlations with insight and TSAs among the patients [42]. Therefore, consideration of DM facets is required to achieve a nuanced understanding of the relationships between DM and TSAs.

In addition to dispositional factors, several demographic variables contribute to MHL and TSAs. Higher education levels are significantly linked to greater MHL and more positive TSAs [43,44]. Age also plays a role, as older adults with chronic conditions may delay seeking support due to higher internalized stigma, lower mental health knowledge, and reduced symptom recognition [45]. Lower-income individuals generally experience more psychological distress while also having lower MHL [46,47]. Additionally, Ziapour et al. [48] found that income was one of the strongest predictors of health literacy among diabetic patients. Gender differences also emerge, with men exhibiting lower MHL than women and being less likely to seek professional help [49]. Married individuals tend to have more positive attitudes toward seeking mental health services compared to single individuals [50]. Given the strong association between demographic factors, MHL, and TSAs, it is crucial to control for these variables when examining the unique contributions of FFMQ facets in this context.

## 2. Current Study

The literature suggests that DM may relate to higher MHL. An enhanced ability to seek out treatment for psychological distress is associated with higher levels of MHL, with the FFMQ facets of Describe, Act with Awareness, and Non-Judgment being particularly influential in this association [35]. To the best of the researchers’ knowledge, only one study to date has examined the relationship between DM and MHL, finding a moderate positive correlation between the constructs among university students. Given that Lo et al. [35] utilized a unidimensional measure of DM, the potentially nuanced relationships between DM facets, MHL, and TSAs have yet to be investigated. As such, it is not clear whether facets interact with mental health knowledge to explain TSAs, given the distressing nature of increased awareness of symptoms. Whether and which of the facets provide incremental validity in explaining MHL and TSAs beyond the variance contributed by demographics alone is also yet to be discovered. Further, it is not clear if, as consistent with the TPB, a sense of self-control (self-efficacy) can explain the relationships between facets and improved TSAs.

In this study, we examined the direct relationships of the FFMQ facets with self-efficacy, MHL, and TSAs, through a secondary dataset that was collected by the first author for their honors thesis [51]. The results of the original study looking at DM’s broad relationship with MHL and TSAs were the rationale for conducting the present study, as significant relationships were found. To understand the relationship better, the present study sought to examine the specific facets and their relationship with MHL and TSAs. We further explored a potential moderator of the relationship between MHL and TSAs. We examined the following hypotheses:

1. All facets will exhibit moderate positive correlations with MHL, TSAs, and general self-efficacy;
2. FFMQ-24’s facets of Describe, Act with Awareness, and Non-Judgment will be significant predictors of MHL over and above demographic variables;
3. FFMQ-24’s facets of Observe, Describe, Act with Awareness, Non-Judgment, and Non-Reactivity will be significant predictors of TSAs over and above demographic variables;
4. Describe, Act with Awareness, and Non-Judgment [33] will moderate the relationship of MHL with TSAs;
5. General self-efficacy will mediate the relationships of Describe, Act with Awareness, and Non-Judgment with TSAs.

### 2.1. Methods

#### 2.1.1. Participants

The current study utilizes a dataset used for the first author’s honors thesis [51]. Participants were recruited online using Amazon Mechanical Turk (AMT) extension’s TurkPrime, a crowdsourcing website, in January 2023 [52]. A power analysis was not run, as it was a secondary dataset. However, prior research demonstrated that a sample of 251 participants was sufficient for a similar research design [53]. There are also several Monte Carlo simulation studies that demonstrate that correlation and regression coefficients stabilize with sample sizes at *n* = 250 [54,55]. As the current study was targeting English-speaking individuals, participant recruitment was limited to English-speaking nations: Australia, Canada, New Zealand, the United Kingdom, and the United States. Inclusion criteria were as follows: (1) at least 18 years of age; (2) an English language proficiency of six or above on a scale from one to ten; and (3) passing the included attention check question. Before inclusion criteria were implemented, a total of 313 respondents completed the survey; however, *n* = 10 were excluded from the sample for low English language proficiency, *n* = 2 were excluded for not responding to the English language proficiency item listed in the demographics questionnaire, and *n* = 2 were excluded for failing the attention check question. The final sample consisted of *N* = 299 participants for all analyses, except the multiple regressions, as one participant had to be excluded due to not disclosing their gender, which was a demographic predictor we used. Table 1 provides a summary of participant demographics. The current study was approved by the first author’s institutional Research Ethics Board (#2022-232).

#### 2.1.2. Measures

**Five-Facet Mindfulness Questionnaire**

The Five-Facet Mindfulness Questionnaire-24 (FFMQ-24) [56] is a 24-item, shortened version of the original 39-item FFMQ-24 developed by Baer et al. [19]. The FFMQ-24 measures DM on five facets: Observe, Describe, Act with Awareness, Non-Judgment of Inner Experience, and Non-Reactivity to Inner Experience. Respondents rated their agreement with each item on a 5-point Likert scale, ranging from 1 (*never or very rarely true*) to 5 (*very often or always true*). An example item includes “*I tell myself that I shouldn’t be feeling the way I’m feeling*”. Upon reversing negatively worded items, higher scores are indicative of high DM levels, and among a study with similar participants (i.e., crowdsourcing recruitment), the FFMQ-24 produced a Cronbach’s alpha of 0.79 [57]. In the present study, Cronbach’s alphas for the facets were as follows: Observe = 0.83, Describe = 0.87, Act with Awareness = 0.87, Non-Judgment = 0.87, and Non-Reactivity = 0.87. The Cronbach alpha for the entire FFMQ-24 scale was 0.90.

**Mental Health Literacy Scale**

The Mental Health Literacy Scale (MHLS) [58] is a self-report measure, consisting of 35 items, that assesses an individual’s knowledge and understanding of different aspects of mental health. An example of an item on the scale is “*To what extent do you think that Personality Disorders are a category of mental illness?*” For questions 1 to 10 and 13 to 15, responses are rated on a 4-point Likert scale, ranging from 1 (*very unlikely*) to 4 (*very likely*). Items 11 and 12 are also rated on a 4-point Likert scale, ranging from 1 (*very unhelpful*) to 4 (*very helpful*). Items 16–28 are rated on a 5-point Likert scale, ranging from 1 (*strongly disagree*) to 5 (*strongly agree*), as well as items 29–35 being rated on a 5-point Likert scale, but with responses ranging from 1 (*definitely unwilling*) to 5 (*definitely willing*). Higher scores are indicative of higher knowledge of mental health and its related concepts. The Cronbach’s alpha values for the 4-point and 5-point Likert scales were 0.84 and 0.89, respectively.

**Mental Help-Seeking Attitudes Scale**

The Mental Help-Seeking Attitudes Scale-9 (MHSAS) [59] is a 9-item scale that measures a participant’s evaluation of mental health professionals and treatments in circumstances where they find themselves experiencing psychological distress. The scale is presented to participants on a 7-point semantic differential scale, and they are prompted with the following phrase: “*If I had a mental health concern, seeking help from a mental health professional would be…*” The scale offers a list of adjectives and their opposites to describe their attitudes, such as “important” or “unimportant” with ratings from zero to three, but only allowing for the participant to pick one option. Higher scores indicate more positive attitudes towards mental health professionals and treatment. The measure has been used extensively in psychology research, as one systematic review on patient-reported outcome measures of TSAs rated the MHSAS as “class A” [60]. Accordingly, the Cronbach’s alpha for the present study was 0.94.

**General Self-Efficacy Scale**

The General Self-Efficacy Scale-10 (GSES) [61] is a 10-item scale that measures a person’s own perceived sense of self-efficacy. The scale was developed to predict capacity to cope with daily life stressors and positive adaptation after adverse life events [60]. An example of an item on the GSES is “*I can always manage to solve difficult problems if I try hard enough*”. The responses are measured on a 4-point Likert scale ranging from 1 (*not at all true*) to 4 (*exactly true*). Higher scores on the GSES indicate greater perceived self-efficacy. The Cronbach’s alpha for the current study was 0.92.

#### 2.1.3. Procedure

Questionnaires were hosted on Qualtrics. Upon viewing the study title and description on TurkPrime, interested participants were directed to click a survey link, which connected them to the study consent procedure and measures. Prior to completing any measures, participants were required to provide their informed consent. Upon completion of study measures, participants were presented with a demographic information form. Besides gathering basic demographic information related to age, gender, income, marital status, education, and ethnic background, we also asked respondents to report whether they had received any formal psychotherapy in the past, if they had ever received a mental health diagnosis, whether they had been involved in a mindfulness-based practice, and their current knowledge levels (from 1 to 10) related to mindfulness. The demographic form also included an attention check item required to pass to be included in the study (i.e., *“What was this survey about? Please do not select “Mindfulness” but instead choose “Other” and type “Psychology” in the text box”*). A debriefing form was provided to participants after the demographics form, and they were automatically compensated USD 2.50 through AMT.

### 2.2. Data Analyses

Prior to running any analyses, all collected data were first scanned for missingness and completion, cleaned, and reverse-scored as appropriate. Skewness and kurtosis values were tabulated to evaluate any violations of assumptions of normalcy, as well as multicollinearity. The study’s data were collected for use in a separate study by the first and last authors; therefore, preliminary Pearson correlations were used as rationale for the current study, as significant associations were found between FFMQ-24’s specific facets of Observe, Describe, Act with Awareness, Non-Judgment, Non-Reactivity, MHL, and TSAs. Though not pertinent to the present study, three independent-sample *t*-tests were conducted to observe nuances in MHL and TSAs between groups that may have inherent differences. Specifically, differences in MHL and TSAs between those who have or have not received a mental health diagnosis, those who have or have not received psychotherapy, and those who do or do not currently engage in mindfulness practices were examined. Two multiple regressions were conducted with demographics (i.e., age, gender, marital status, income, and education) in the first block, and FFMQ-24 facets in the second block (i.e., Observe, Describe, Act with Awareness, Non-Judgment, and Non-Reactivity). Subsequently, we conducted five exploratory moderation analyses using the Hayes [62] PROCESS SPSS v. 28 macro. The five moderators were the five facets of the FFMQ-24. The moderation analyses, Model 1, used 5000-sample bootstrapping, and significant interactions were followed up with conditional effects examining the strength of the relationships between scores on the MHLS and MHSAS at −1 SD of the moderator, mean of the moderator, and +1 SD of the moderator. The critical alpha for these moderations was Bonferroni-adjusted to 0.05/5 or 0.01.

Finally, we conducted five exploratory mediation analyses using PROCESS (Model 4) [62] in SPSS v. 28 to examine whether general self-efficacy mediated the relationship between various facets of mindfulness and attitudes toward mental help-seeking. Separate mediation models were estimated for each mindfulness facet (i.e., Observe, Describe, Act with Awareness, Non-Judgment, Non-Reactivity) as predictors (X), with general self-efficacy scores (GSESs) serving as the mediator (M) and scores on the MHSAS as the outcome (Y).

For each model, ordinary least squares regression was used to estimate the path from the predictor to the mediator (a path), from the mediator to the outcome (b path), and the direct effect of the predictor on the outcome (c′ path), controlling for the mediator. The total effect (c path) was also computed for each mindfulness facet. Indirect effects were determined using 5000 bootstrap samples to construct bias-corrected 99% confidence intervals, with mediation deemed significant when the confidence interval did not include zero. In light of multiple comparisons across the five facets, a Bonferroni-adjusted alpha level of 0.01 was applied to interpret the significance of indirect (mediation) effects.

## 3. Results

### 3.1. Demographics

The final sample consisted of *N* = 299 participants (*N* = 298 for multiple regressions; see participant section above), *M_age_* = 41.02, and 49.7% identifying as a cis woman. *n* = 1 preferred not to disclose gender, and *n* = 14 did not meet the inclusion criteria. For all regressions, those who identified as a trans man (*n* = 1) were collapsed into man, those who identified as other and specified identifying as a woman or female (*n* = 2) were collapsed into woman, and those who identified as a man or male (*n* = 2) were collapsed into man. For the multiple regressions, women (vs. men) and marriage status (45.5%) vs. single, divorced/separated, or widowed were the reference group. The highest level of education was not dummy-coded, as it was hierarchical in the nature of the degree (e.g., 1 = no high school diploma; 2 = high school diploma). In the current sample, *n* = 80 participants (26.8%) reported receiving psychotherapy, while *n* = 207 (69.2%) reported never receiving any form of psychotherapy in the past (and *n* = 12 not disclosing their previous psychotherapy exposure). People who reported receiving any form of psychotherapy also reported higher scores on the MHLS (*t*(285) = 7.51, *p* < 0.001 (Cohen’s *d* = 0.99)) and higher scores on the MHSAS (*t*(285) = 2.10, *p* = 0.04, (Cohen’s *d* = 0.28)). Further, *n* = 85 participants (28.5%) reported current engagement in a mindfulness meditation practice, while *n* = 210 (70.2%) did not, and *n* = 4 (1.3%) preferred not to disclose. On a 10-point Likert scale, where 1 is not at all knowledgeable and 10 is very knowledgeable about mindfulness, the average mindfulness knowledge was *M* = 4.74 (*SD* = 2.86). Table 1 provides a summary of pertinent demographics.

#### Zero-Order Correlations Among Study Measures

Zero-order correlation coefficients of scores on the five FFMQ-24 facets, MHLS, the MHSAS, and GSES are summarized in Table 2. As can be observed, Describe was positively associated with MHLS (*r* = 0.17, *p* < 0.01), MHSAS (*r* = 0.18, *p* < 0.01), and GSES (*r* = 0.50, *p* < 0.01). Non-Reactivity was not significantly related to MHLS but showed significant positive relationships with MHSAS (*r* = 0.16, *p* < 0.01) and GSES (*r* = 0.56, *p* < 0.01). Non-Judgment was correlated modestly with MHLS (*r* = 0.12, *p* < 0.05), MHSAS (*r* = 0.14, *p* < 0.05), and GSES (*r* = 0.24, *p* < 0.01). Observe was not significantly linked to MHLS but showed significant positive associations with MHSAS (*r* = 0.13, *p* < 0.05) and GSES (*r* = 0.24, *p* < 0.01). Finally, Act with Awareness demonstrated small-to-moderate correlations with MHLS (*r* = 0.13, *p* < 0.05), MHSAS (*r* = 0.23, *p* < 0.01), and GSES (*r* = 0.30, *p* < 0.01). There were significant relationships among the three outcome measures, with MHLS being positively associated with MHSAS (*r* = 0.37, *p* < 0.01) and GSES (*r* = 0.13, *p* < 0.05). Finally, MHSAS was correlated with GSES (*r* = 0.20, *p* < 0.01).

### 3.2. Independent-Sample t-Tests

#### 3.2.1. Mental Health Diagnosis

An independent-sample *t*-test was conducted to examine whether there were significant differences between those who had received a mental health diagnosis and those who had not. A detailed breakdown of each *t*-test is presented in Table 3. There was a significant difference noted between the two groups for MHLS, but Levene’s Test for equality of variances was significant at the 0.01 alpha level (*F*(284, 247.70) = 14.924, *p* = <0.001), indicating that the assumption of equal variances was violated. The results of the *t*-test assuming unequal variances (Welch’s *t*-test) were reported: *t*(247.70) = 7.316, *p* < 0.001, *d* = 0.82, 95% CI [9.22, 16.02]. Those with a mental health diagnosis scored significantly higher on the measure of MHLS (*M* = 131.41; *SD* = 11.90) than those without a prior mental health diagnosis (*M* = 118.80; *SD* = 16.78). This suggests that those with a mental health diagnosis had higher MHL scores.

There was a significant difference between the same two groups for the GSES: *t*(284) = −3.691, *p* < 0.001, *d* = −0.47, 95% CI [−3.92, −1.19]. Those with a mental health diagnosis scored lower (*M* = 28.17; *SD* = 5.96) than those without (*M* = 30.72; *SD* = 5.26). The findings implied that those with a mental health diagnosis have lower general self-efficacy.

In terms of DM facets, there were significant differences noted between the groups for Act with Awareness and Non-Reactivity. For Act with Awareness, those with a mental health diagnosis had lower Act with Awareness capabilities (*M* = 16.63; *SD* = 4.44), such that *t*(284) = −2.663, *p* = 0.008, *d* = −0.34, 95% CI [−2.69, −0.40]. These results suggested that those with a mental health diagnosis display less acting with awareness in everyday situations.

There was also a significant difference between the groups for Non-Reactivity; however, Levene’s Test for equality of variances was significant at the 0.05 alpha level: *F*(284, 158.243) = 5.046, *p* = 0.025. The results of the *t*-test assuming unequal variances (Welch’s *t*-test) are reported: *t*(158.243) = −4.354, *p* < 0.001, *d* = −0.58, 95% CI [−3.59, −1.35]. Those with a mental health diagnosis scored lower on Non-Reactivity (*M* = 14.56; *SD* = 4.76) than those without a mental health diagnosis (*M* = 17.04; *SD* = 3.97). These findings suggest that those with a mental health diagnosis may have a stronger tendency to be reactive to thoughts and emotions.

#### 3.2.2. Prior Psychotherapy

Independent-sample *t*-tests were conducted to see whether there were significant differences between participants who had a history of psychotherapy and those who did not. A detailed breakdown of each *t*-test is shown in Table 4. A significant difference was noted between those who had received psychotherapy and those who did not for MHLS; however, Levene’s Test for equality of variances was significant at the 0.01 alpha level: *F*(285, 210.44) = 16.336, *p* = <0.001. Therefore, the results of the *t*-test assuming unequal variances (Welch’s *t*-test) are reported: *t*(210.44) = 8.857, *p* < 0.001, *d* = 0.99, 95% CI [11.58, 18.21]. Those who had received psychotherapy scored significantly higher on the MHLS (*M* = 133.65; *SD* = 11.09) than those who had never received psychotherapy (*M* = 118.76; *SD* = 16.34). Results indicated that higher MHL may be associated with higher engagement in psychotherapy.

The only facet to demonstrate a significant difference between the two groups was Describe, at the 0.05 level: *t*(285) = 2.017, *p* = 0.045, *d* = 0.27, 95% CI [0.03, 2.33]. Participants who had received psychotherapy had higher rates of describing internal experiences (*M* = 18.70; *SD* = 4.40) compared to those without prior exposure to psychotherapy (*M* = 17.52; *SD* = 4.45). These results suggested that participation in psychotherapy is associated with enhanced abilities in describing internal experiences.

There was a significant difference between the two groups for MHSAS; however, Levene’s Test for equality of variances was significant at the 0.05 alpha level: *F*(285, 160.242) = 4.765, *p* = 0.03. The results of the *t*-test assuming unequal variances (Welch’s *t*-test) are reported: *t*(160.242) = 2.213, *p* = 0.03, *d* = 0.28, 95% CI [0.34, 5.90]. Participants who had received psychotherapy previously scored higher on the MHSAS (*M* = 50.63; *SD* = 10.33) than those who had not (*M* = 47.51; *SD* = 11.61), suggesting that higher TSAs may be associated with higher engagement in psychotherapy.

#### 3.2.3. Current Mindfulness Meditation Practice

Independent-sample *t*-tests were conducted to examine whether there were any significant differences between those who currently have a mindfulness practice and those who do not. A detailed breakdown of each *t*-test is shown in Table 5. A significant difference between the two groups for MHLS was found; however, Levene’s Test for equality of variances was significant at the 0.01 alpha level: *F*(293, 129.752) = 9.163, *p* = 0.003. The results of the *t*-test assuming unequal variances (Welch’s *t*-test) are reported: *t*(129.752) = −2.476, *p* = 0.007, *d* = −0.35, 95% CI [−10.18, −1.14]. Those with a current mindfulness meditation practice scored lower on the MHLS (*M* = 118.92; *SD* = 18.77) than those without one (*M* = 124.58; *SD* = 15.03).

Scores on the FFMQ facet of Observe were significantly different between groups: *t*(293) = 3.904, *p* < 0.001, *d* = 0.50, 95% CI [0.78, 2.38]. Participants with a mindfulness meditation practice scored higher on the Observe facet (*M* = 16.24; *SD* = 2.68) than those without a mindfulness practice (*M* = 14.65; *SD* = 3.33).

### 3.3. Multiple Regressions

#### 3.3.1. Mental Health Literacy Scale

A hierarchical multiple regression was run with demographic variables (i.e., age, gender, marital status, income, and education) in the first block, and all FFMQ-24 facets (Observe, Describe, Act with Awareness, Non-Judgment, and Non-Reactivity) were entered in the second block. The overall model with both sets of predictors was significant: *F*(10, 297) = 4.76, *p* < 0.001. The first model, with demographic predictors, was significant: *F*(5, 292) = 6.97, *R*^2^ = 0.11, *p* < 0.001. When all facets were added in the second block, they contributed to significant variation in MHLS scores: Δ*F*(5, 287) = 2.39, *R*^2^ = 0.14, Δ*R*^2^ = 0.11, *p* < 0.04. However, the only significant predictors in the model were Describe (*t*(5,287) = 2.09, *p* < 0.04) and Non-Reactivity (*t*(5,287) = −2.51, *p* < 0.01) despite Non-Reactivity not having a prior association with MHLS scores. Demographic predictors included age, being married, and education level. Seemingly, higher scores on Describe, as well as older age, being married, and education, predicted MHLS scores. Regression coefficients are presented in Table 6.

#### 3.3.2. Mental Help-Seeking Attitudes Scale

A second hierarchical regression was conducted to evaluate whether FFMQ-24 facets could predict MHSAS scores over demographic variables. In the first step, demographic variables (i.e., age, gender, marital status, income, education) were added, and the FFMQ-24 facets of Observe, Describe, Act with Awareness, Non-Judgment, and Non-Reactivity were added in the second block. The overall model was significant: *F*(10, 297) = 2.79, *p* < 0.003. The first model with only demographics as predictors was not significant: *F*(5, 292) = 0.86, *R*^2^ = 0.01, *p* = 0.52. However, when FFMQ-24 facets were included, the model became significant: Δ*F*(5, 287) = 4.67, *R*^2^ = 0.09, Δ*R*^2^ = 0.06, *p* < 0.001. No demographics were significant predictors, and only one FFMQ-24 facet was a significant predictor: Act with Awareness, *t*(5,287) = 2.92, *p* < 0.004. Regression coefficients are presented in Table 7.

### 3.4. Moderation Analyses

As planned, five Bonferroni-adjusted PROCESS [62] moderation analyses were conducted to determine whether any of the FFMQ-24 facets moderated the relationship between the MHLS and MHSAS. The only variable that survived the Bonferroni-adjusted alpha was Non-Reactivity. The overall regression model was significant: *F*(3, 295) = 23.09, *p* < 0.000. The interaction of Non-Reactivity and MHLS scores predicted incremental variance in MHSAS scores over and above the main effects of Non-Reactivity and MHLS scores: *F*(1, 295) = 7.47, Δ*R*^2^ = 0.02, *p* < 0.007. Conditional effects indicate that at mean and +1 SD, the effects are significant. At mean (Non-Reactivity = 0.0000), the effect was *B* = 0.27, *SE* = 0.04, *p* < 0.0001, and at +1 SD (Non-Reactivity = 4.4105), the effect was *B* = 0.37, *SE* = 0.05, *p* < 0.0001. However, at −1 SD, it was approaching significance (Non-Reactivity = −4.4105), with an effect of *B* = 0.16, *SE* = 0.05, *p* < 0.002. Altogether, these results indicate that as Non-Reactivity scores increase, the relationship between MHLS and MHSAS scores also increases, confirming that higher levels of Non-Reactivity intensify the relationship between MHLS and MHSAS.

Act with Awareness appeared to have a marginally significant moderation effect. The overall regression model was significant: *F*(3, 295) = 21.72, *p* < 0.001. The interaction effect was significant at the 0.05 alpha level (*F*(1, 295) = 4.11, Δ*R*^2^ = 0.01, *p* < 0.04) but did not survive the corrected alpha of 0.01. A summary of all five moderation analysis coefficients is presented in Table 8.

### 3.5. Mediation Analyses

Five mediation analyses examining whether GSES scores explained the relationship between each of the FFMQ-24 facets and MHSAS were conducted as planned. A summary of these analyses is presented in Table 9. These analyses were also Bonferroni-adjusted to a critical alpha of 0.01. The GSES demonstrated full mediation of the relationship between Observe and the MHSAS, and full mediation of the relationship between Non-Judgment and the MHSAS. No other mediation analyses survived the Bonferroni-corrected critical alpha.

## 4. Discussion

Behaviors related to treatment-seeking are influenced by attitudes, an understanding of the source of the illness [63], awareness of treatment options, and perceived severity of the distress, all of which are key components in MHL [64]. The present study sought to determine what facets of the FFMQ-24 were most strongly predictive of MHL and treatment-seeking, as well as which facets may be able to moderate and are involved in the mediation of the relationship between MHL and treatment-seeking. The current study yields promising results to the field of mental health promotion and provides a rationale for mindfulness being integrated into educational systems and mental health treatment/prevention programs.

### 4.1. Demographics

Demographics have consistently been found to correlate with MHL and TSAs [43,44,45,47,48]. In the present study, participants who reported receiving any form of psychotherapy reported higher MHL and TSAs; accordingly, these outcomes seem to be a valid indicator of treatment-seeking behavior. This is consistent with previous findings, which showed that participants who received therapy had higher levels of MHL compared to those not in therapy [65]. Relatedly, attitudes towards treatment were regarded as more positive, and acceptance was higher for those who had received treatment for psychological distress or had a close relative with treatment experience [44]. A longitudinal study found that positive attitudes towards mental health care and higher MHL predicted the use of psychotherapy at the 6-month follow-up, controlling for sociodemographic variables and symptomatology [66].

Consistent with the evidence reviewed, in the present study, we found that age, marital status, and education emerged as significant predictors of MHL. The results showed that older, married participants and those reporting higher education have lower rates of MHL. While previous studies found older age to be associated with lower MHL [45], the finding that being married and having higher education were correlated with lower MHL was inconsistent with the existing literature [43,44,50]. Studies on relationship statuses have found evidence that those identifying as single report more psychological symptoms [67], perceived stress and loneliness [68], as well as lower perceived social support and life satisfaction [69]; however, married individuals demonstrated greater life satisfaction, lower stress, and less depression [70]. This may be in line with prior results that exposure to therapeutic services increases MHL and treatment-seeking, as depending on life satisfaction and psychological health, married individuals may not experience psychological distress as often.

The Dunning–Kruger effect [71], or the metacognitive bias associated with overconfidence in one’s abilities, may provide insights regarding education’s paradoxical relationship with MHL. Dunning and Kruger [71] proposed that incompetence hinders individuals’ ability to recognize their mistakes and wrongful conclusions. Competence may be assumed to be correlated with education; however, this is not always true, as in the case of the present study. Heightened perception of competence may result in individuals overlooking their capacity to make mistakes in areas that may not be within their expertise. The current results showed a negative relationship between education and MHL rates, which is often seen in health literacy, a concept closely related to MHL [72,73]. Those with high self-rated health knowledge scored lower on health literacy measures compared to those with higher health literacy, and demonstrated more overconfidence [72]. This overconfidence was related to poorer health behaviors and outcomes [72]. In the present study, this may explain the seemingly paradoxical relationship between higher education and lower MHL.

When separating the present sample into groups based on pre-existing variables, significant differences were noted. MHL specifically was associated with having a mental health diagnosis, a history of psychotherapy, and current mindfulness meditation practices. Those with a mental health diagnosis or a history of psychotherapy had higher rates of MHL than those who did not. This is likely due to the nature of receiving a diagnosis and psychotherapy. When diagnoses are provided, it is likely that the practitioner will provide psychoeducation on that diagnosis and possible treatments available. It is also possible that those with higher MHL are more likely to seek formal diagnoses for their distress. Those with a current meditation practice had lower rates of MHL compared to those without. This is not consistent with prior research that found that mindfulness meditation was positively associated with emotional intelligence [74]. It is possible, however, that those who are engaging in meditation are seeking to improve their mindfulness skills and hence their MHL. A more nuanced understanding would be benefitted by follow-up questions in the present study regarding how long participants had been engaging in mindfulness, the frequency of their practice, the types of meditation practiced, and what instructions or training were followed.

TSAs were higher in those who had received psychotherapy. This finding is consistent with the literature, as those with previous exposure to psychotherapy are more likely to have sought treatment. Self-efficacy was significantly lower in participants who had received a mental health diagnosis. Consistent with TPB, having higher self-efficacy may help in the initiation of treatment. It is also plausible that treatment may increase self-efficacy through teaching coping skills. These findings do corroborate other findings, as one study found that individuals with low self-efficacy were more likely to have a mental health condition [75].

In terms of facets, those with a mental health diagnosis had lower rates of the Act with Awareness facet. This may be explained by prior research indicating that Act with Awareness is inversely related to psychological distress symptoms [31]. A psychological disorder is diagnosed through exploration of an individual’s symptoms; therefore, it is plausible to assume that those with a diagnosis may have lower tendencies to act with awareness, as explained by Carpenter et al. [31].

Those with a current mindfulness meditation practice were found to have higher scores on the facet of Observe than those who did not have a current practice. The nature of mindfulness meditation inherently requires an ability to attend to feelings, thoughts, and sensations (Observe). Observe has been found to be able to differentiate between meditators and non-meditators [76], and predicts self-efficacy [30]. In the current context, mindfulness meditations require individuals to stick to a regimen of frequent practice, which may indicate participants requiring higher self-efficacy skills.

### 4.2. Facet Predictors

In the current study, and consistent with the hypotheses, the facet of Describe was a robust predictor of MHL. This finding is aligned with the finding that the ability to name or label internal experiences may increase recognition of mental health and its related resources [77]. This is also consistent with extant research [31,33]. A meta-analysis identified a strong relationship between Describe and the ability to label emotional reactions against physical reactions, promoting adaptive acknowledgment and regulation of negative affect [31]. Also in the present study, we found that Non-Reactivity had an inverse relationship with MHL after controlling for the effects of other facets, demonstrating a potential suppression effect. This suggests that, in the absence of other facets of mindfulness, being highly non-reactive to internal experiences may reduce the drive to seek out information or perceive a need for seeking out mental health resources. Low or no aversive feelings in reaction to symptoms, as consistent with Non-Reactivity, may explain why, when all facets have been controlled, Non-Reactivity may be associated with reduced drive to seek information regarding mental health symptoms. Consistent with our hypotheses, Act with Awareness was the only significant predictor of treatment-seeking once demographics had been controlled for. Present-focused awareness of psychological distress may make it more likely for individuals to consider or pursue appropriate and professional services rather than stigma coping [42]. These findings correlate with prior research that found a negative relationship between Act with Awareness and alexithymia [33], and its positive relationship with self-efficacy [30].

### 4.3. Facet Moderators

Non-Reactivity strengthened the positive association between MHL and TSAs and behaviors. At higher levels of Non-Reactivity, being informed about mental health appeared to be more likely to translate into positive treatment-seeking attitudes, which is consistent with prior research [42]. The results of the current study suggest that, while Non-Reactivity may reduce the drive to seek out mental health information, individuals who already possess adequate baseline information regarding mental health may be more moved toward appropriate treatment-seeking when this information is combined with higher levels of Non-Reactivity to inner experiences.

### 4.4. Facet Mediators

The GSES mediated the relationship between Observe and TSAs and behaviors, as well as that between Non-Judgment and TSAs and behaviors. This is in support of the TPB [22], which suggests that some dispositional factors might create a better sense of self-efficacy over behavior. Higher self-efficacy then spills over into more positive attitudes, moving individuals toward appropriate action and change. Mindfulness includes qualities that strengthen intention–behavior relationships, strengthening self-control abilities [78], and characteristics of lower mindfulness levels weaken the intention–behavior relationships [17,78,79]. In the current study, it appeared that both Observe and Non-Judgment may increase one’s sense of control over mental health challenges and capacity to seek treatment. Non-Judgment has been construed as accepting without judgment in earlier research and was found to be related to greater coping self-efficacy [80]. Coping self-efficacy mediated the relationship between Non-Judgment and emotional regulation, which is believed to be the result of mindfulness fostering a greater sense of self-control [80]. It is likely that individuals higher in self-efficacy are able to observe internal events and respond appropriately, which may facilitate treatment-seeking.

### 4.5. Practical Applications

Understanding the precise mechanisms at play between DM, MHL, and TSAs has important implications both for individuals and for healthcare systems. The results of the current study demonstrated that each facet of DM plays a unique role in MHL and TSAs. The facets can be targeted within existing interventions to improve MHL and help-seeking intentions to improve early initiation of therapy and hence prevent additional individual and healthcare system burdens. Further, DM may be incorporated into preventative public health measures against psychological distress in efforts to increase MHL and TSAs. Educational systems may benefit from introducing DM to children and adolescents, as in one population-based study, adolescents with moderate to severe depression were more likely to have low MHL [81]. This suggests that enhanced MHL may serve as a protective factor against developing depressive symptoms.

### 4.6. Strengths and Limitations

The present study yielded a relatively large sample size from several countries, and had an even split amongst genders, allowing for generalization across the countries and genders represented in the current study. The measures used have excellent psychometric properties and are commonly used measures of the constructs. Further, the current study was theory-driven (TPB). This theory-drivenness focused on increasing health promotion allows for easier implementation into current practices and makes the development of appropriate MHL interventions more accessible.

With that, there were several notable limitations that pave the way for future studies. First, measures were administered solely in English, and most participants were white (77.3%). Accordingly, the generalizability of the results to other ethnoracial categories is limited. MHL and TSAs are heavily dependent on culture, as a meta-analysis noted that certain facets differed in impact on affective symptoms across Eastern and Western cultures [31]. Future research should extend to examine how specific facets may be associated with MHL and TSAs and whether these differ by culture. Identifying these differences will better help clinicians tailor interventions to clients from cultures that differ from their own. Second, the study had a cross-sectional design, which does not allow for causal inferences to be drawn and presents questions related to the replicability of some of the analyses (e.g., mediation). Third, mindfulness has several operationalizations, so our results may not corroborate other operationalizations of mindfulness. Lastly, the use of TurkPrime as a recruitment method has inherent sampling biases, a noted weakness in the present study. In particular, ethnicity, income distribution, education, and religion may be significantly related to the outcome and independent measures. However, the present results provide a foundation for future research to further explore these associations between DM and its facets with MHL and TSAs to obtain a more nuanced understanding.

## 5. Conclusions

The present study’s findings demonstrated the relationships between DM and important clinical outcomes related to literacy and treatment-seeking. DM does not appear to function as a unitary concept, and interventions designed to improve treatment-seeking should focus specifically on cultivating specific facets of mindfulness, which may work to enhance MHL and/or treatment-seeking attitudes alone or in interaction with other facets or constructs. Given the results in the current study, and assuming successful replication, MHL interventions should focus on cultivating the Observe facet of mindfulness, which appears to work through general self-efficacy to increase treatment-seeking attitudes. Interventions should balance Non-Reactivity with increases in MHL to foster better attitudes toward seeking help. Finally, cultivation of facets such as Non-Judgment also appears to have a direct relationship with a sense of control over health outcomes, and hence works through self-efficacy to improve attitudes toward help-seeking. Cultivating self-efficacy seems to be key for individuals high in Observe and Non-Judgment, which may facilitate translation into positive attitudes and adaptive treatment-seeking behavior. All in all, this study demonstrated that the concept of dispositional mindfulness is multifaceted, and should be considered as such for a more granular understanding of literacy and health-related attitudes.

## Figures and Tables

**Table 1:** Summary of pertinent demographics.

Total Sample (*n*)		*N* = 299
Age	**M**	**SD**
	41.04	11.62
Gender	*n*	%
Cis woman	148	49.5
Cis man	145	48.5
Other	6	2.0
Ethnicity	*n*	%
Black	25	8.4
East Asian	11	3.7
Latinx	14	4.7
Middle Eastern	1	0.3
Southeast Asian	10	3.3
White	231	77.3
Other racial categories	6	2.0
Marital Status	*n*	%
Single, never married	123	41.1
Married	136	45.5
Separated/Divorced	35	11.7
Widowed	5	1.7
Children	*n*	%
Yes	144	48.2
No	154	51.5
Approximate Annual Income (CAD)	*n*	%
Unemployed/No yearly income	13	4.3
10,000–30,000	84	28.1
31,000–50,000	66	22.1
51,000–75,000	59	19.7
76,000–99,000	40	13.4
100,000 and over	34	11.4
Other	3	1.0
Highest Level of Education	*n*	%
No degree, certificate, or diploma	5	1.7
Secondary (high) school diploma or equivalent	60	20.1
Trades certificate or diploma	14	4.7
Other non-university certificate or diploma	14	4.7
University certificate or diploma below bachelor level	28	9.4
Bachelor’s degree	121	40.5
Master’s degree	45	15.1
Doctorate	6	2.0
Other	6	2.0
Religion/Belief System	*n*	%
Christianity	157	52.5
Atheism	50	16.7
Agnosticism	61	20.4
Other	31	10.4
First Language	*n*	%
English	294	98.3
Other	5	1.7
Prior Mental Illness Diagnosis	*n*	%
Yes, please specify	94	31.4
No	192	64.2
Prefer not to disclose	13	4.3
Prior Psychotherapy	*n*	%
Yes	80	26.8
No	207	69.2
Prefer not to disclose	12	4.0
Mindfulness/Mindfulness-Based Intervention Knowledge (0–10 scale)	*n*	%
0	15	5.0
1	40	13.4
2	29	9.7
3	26	8.7
4	23	7.7
5	41	13.7
6	36	12.0
7	36	12.0
8	23	7.7
9	9	3.0
10	21	7.0
Prior Mindfulness-Based Intervention Participation	*n*	%
Yes	49	16.4
No	244	81.6
Prefer not to disclose	6	2.0
Current Mindfulness Practices	*n*	%
Yes	85	28.4
No	210	70.2
Prefer not to disclose	4	1.3
Current Anti-Depressant Medication	*n*	%
Yes	43	14.4
No	254	84.9
Prefer not to disclose	2	0.7

**Table 2:** Pearson correlations between pertinent study variables.

Variable	1	2	3	4	5	6	7	8
1. OB	1.0	-						
2. DS	0.33 **	1.0	-					
3. AA	0.17 **	0.50 **	1.0	-				
4. NJ	−0.01	0.43 **	0.51 **	1.0	-			
5. NR	0.20 **	0.32 **	0.23 **	0.30 **	1.0	-		
6. MHLS	0.08	0.17 **	0.13 *	0.12 *	−0.07	1.0	-	
7. MHSAS	0.13 *	0.18 **	0.23 **	0.14 *	0.16 **	0.37 **	1.0	-
8. GSES	0.24 **	0.50 **	0.30 **	0.23 **	0.56 **	0.13 **	0.20 **	1.0

*Note:* 1. OB = Observe facet; 2. DS = Describe facet; 3. AA = Act with Awareness facet; 4. NJ = Non-Judgment facet; 5. NR = Non-Reactivity facet; 6. MHLS = Mental Health Literacy Scale; 7. MHSAS = Mental Help-Seeking Attitudes Scale; 8. GSES = General Self-Efficacy Scale; all values rounded up to one decimal place. ** = significant at the 0.01 level; * = significant at the 0.05 level.

**Table 3:** Independent-sample *t*-tests for participants with a mental health diagnosis and those without.

**OB**	*N*	*M*	*SD*	*F*	*Sig*	*t*	*df*	*p*	*Lower*	*Upper*	*D*
Yes	94	14.98	3.18								−0.05
No	192	15.14	3.28								
*Equal Variances Assumed*				0.889	0.347	−0.396	284	0.692	−0.97	0.64	
**DS**											−0.17
Yes	94	17.36	4.76								
No	192	18.12	4.28								
*Equal Variances Assumed*				3.031	0.083	−1.356	284	0.176	−1.86	0.34	
**AA**											−0.34
Yes	94	16.63	4.44								
No	192	18.18	4.71								
*Equal Variances Assumed*				1.145	0.286	−2.663	284	**0.008** **	−2.69	−0.40	
**NJ**											−0.18
Yes	94	15.27	4.84								
No	192	16.14	4.78								
*Equal Variances Assumed*				0.000	0.993	−1.440	284	0.151	−2.06	0.32	
**NR**											−0.58
Yes	94	14.56	4.76								
No	192	17.04	3.97								
*Equal Variances Not Assumed*				5.046	**0.025** *	−4.354	158.243	**<0.001** **	−3.59	−1.35	
**MHSAS**											0.17
Yes	94	49.80	10.76								
No	192	47.92	11.57								
*Equal Variances Assumed*				4.018	0.046	1.318	284	0.189	−0.93	4.68	
**GSES**											−0.47
Yes	94	28.17	5.96								
No	192	30.72	5.26								
*Equal Variances Assumed*				0.640	0.425	−3.691	284	**<0.001** **	−3.92	−1.19	
**MHLS**											0.82
Yes	94	131.41	11.91								
No	192	118.80	16.78								
*Equal Variances Not Assumed*				14.924	**<0.001** **	7.316	247.700	**<0.001** **	9.22	16.02	

*Note:* 1. OB = Observe facet; 2. DS = Describe facet; 3. AA = Act with Awareness facet; 4. NJ = Non-Judgment facet; 5. NR = Non-Reactivity facet; 6. MHLS = Mental Health Literacy Scale; 7. MHSAS = Mental Help-Seeking Attitudes Scale; 8. GSES = General Self-Efficacy Scale; all values rounded up to one decimal place. ** = significant at the 0.01 level; * = significant at the 0.05 level.

**Table 4:** Independent-sample *t*-test for participants with a history of psychotherapy and those without.

**Observe**	*N*	*M*	*SD*	*F*	*Sig*	*t*	*df*	*p*	*Lower*	*Upper*	*d*
Yes	80	15.41	3.19								0.14
No	207	14.97	3.27								
*Equal Variances Assumed*				0.003	0.953	1.032	285	0.303	−0.40	1.28	
**Describe**											0.27
Yes	80	18.70	4.40								
No	207	17.52	4.45								
*Equal Variances Assumed*				0.002	0.964	2.017	285	0.045	0.03	2.33	
**Act with Awareness**											0.07
Yes	80	17.86	4.47								
No	207	17.55	4.76								
*Equal Variances Assumed*				0.906	0.342	0.506	285	0.614	−0.90	1.53	
**Non-Judgment**											0.06
Yes	80	16.05	5.14								
No	207	15.75	4.68								
*Equal Variances Assumed*				1.435	0.232	0.475	285	0.635	−0.95	1.55	
**Non-Reactivity**											−0.19
Yes	80	15.60	4.60								
No	207	16.43	4.30								
*Equal Variances Assumed*				0.388	0.534	−1.438	285	0.151	−1.97	0.31	
**MHSAS**											0.28
Yes	80	50.63	10.33								
No	207	47.51	11.61								
*Equal Variances Not Assumed*				4.765	**0.03** *	2.213	160.242	**0.036** *	0.20	6.04	
**GSES**											−0.025
Yes	80	29.75	5.50								
No	207	29.89	5.67								
*Equal Variances Assumed*				0.089	0.765	−0.188	285	0.851	−1.60	1.32	
**MHLS**											0.99
Yes	80	133.65	11.09								
No	207	118.76	16.34								
*Equal Variances Not Assumed*				16.336	**<0.001** **	8.857	210.44	**<0.001** **	10.99	18.80	

*Note:* 1. OB = Observe facet; 2. DS = Describe facet; 3. AA = Act with Awareness facet; 4. NJ = Non-Judgment facet; 5. NR = Non-Reactivity facet; 6. MHLS = Mental Health Literacy Scale; 7. MHSAS = Mental Help-Seeking Attitudes Scale; 8. GSES = General Self-Efficacy Scale; all values rounded up to one decimal place. ** = significant at the 0.01 level; * = significant at the 0.05 level.

**Table 5:** Independent-sample *t*-test for participants with a current mindfulness meditation practice and those without.

**Observe**	*N*	*M*	*SD*	*F*	*Sig*	*t*	*df*	*p*	*Lower*	*Upper*	*d*
Yes	85	16.24	2.68								0.50
No	210	14.65	3.33								
*Equal Variances Assumed*				3.427	0.065	3.904	293	**<0.001** **	0.78	2.38	
**Describe**											0.03
Yes	85	17.95	4.37								
No	210	17.81	4.51								
*Equal Variances Assumed*				0.292	0.589	0.250	293	0.803	−0.99	1.27	
**Act with Awareness**											−0.10
Yes	85	17.32	4.60								
No	210	17.8	4.66								
*Equal Variances Assumed*				0.045	0.832	−0.807	293	0.420	−1.66	0.69	
**Non-Judgment**											0.04
Yes	85	15.96	4.90								
No	210	15.76	4.83								
*Equal Variances Assumed*				0.038	0.845	0.333	293	0.739	−1.02	1.43	
**Non-Reactivity**											0.13
Yes	85	16.54	4.20								
No	210	15.97	4.52								
*Equal Variances Assumed*				1.496	0.222	1.008	293	0.314	−0.55	1.70	
**MHSAS**											−0.02
Yes	85	48.29	10.50								
No	210	48.50	11.82								
*Equal Variances Assumed*				0.441	0.507	−0.143	293	0.886	−3.11	2.69	
**GSES**											0.19
Yes	85	30.52	5.05								
No	210	29.44	5.88								
*Equal Variances Assumed*				1.142	0.286	1.478	293	0.141	−0.36	2.51	
**MHLS**											−0.35
Yes	85	118.92	18.77								
No	210	124.58	15.03								
*Equal Variances Not Assumed*				9.163	**0.003** **	−2.476	129.752	**0.015** *	−10.18	−1.14	

*Note:* 1. OB = Observe facet; 2. DS = Describe facet; 3. AA = Act with Awareness facet; 4. NJ = Non-Judgment facet; 5. NR = Non-Reactivity facet; 6. MHLS = Mental Health Literacy Scale; 7. MHSAS = Mental Help-Seeking Attitudes Scale; 8. GSES = General Self-Efficacy Scale; all values rounded up to one decimal place. ** = significant at the 0.01 level; * = significant at the 0.05 level.

**Table 6:** Demographics and dispositional mindfulness facets predicting mental health literacy.

		B	SE B	β	t
**Model 1 (Demographics): *R =* 0.34, *R*** **^2^ = 0.11, *F* for change in *R*** ^2^ = 6.97 **					
	Constant	116.20	4.12		28.21
	Age	0.25	0.08	0.17 **	3.10
	Women ref	3.43	1.86	0.11	1.84
	Married	−6.82	2.08	−0.21 **	−3.28
	Education	−1.28	0.43	−0.19 **	−3.01
	Yearly Income	1.45	0.73	0.13 *	2.00
**Model 2 (Demographics and Predicting Factors): *R* = 0.38, *R*** **^2^ = 0.14, Δ*R*** **^2^ = 0.04, *F* for change in *R*** ^2^ = 2.39 *					
	Constant	112.00	6.46		17.3
	Age	0.21	0.08	0.15 **	2.58
	Women ref	2.38	1.88	0.07	1.26
	Married	−6.85	2.09	−0.21 **	−3.27
	Education	−1.19	0.43	−0.17 **	−2.78
	Yearly Income	1.39	0.73	0.13	1.92
	OB	0.18	0.31	0.04	0.59
	DS	0.55	0.26	0.15 *	2.09
	AA	−0.05	0.25	−0.02	−0.21
	NJ	0.21	0.23	0.06	0.89
	NR	−0.57	0.23	−0.15 *	−2.51

*Note:* OB = Observe facet; DS = Describe facet; AA = Act with Awareness facet; NJ = Non-Judgment facet; NR = Non-Reactivity facet. All values have been rounded up to one decimal place. ** = significant at the 0.01 level; * = significant at the 0.05 level.

**Table 7:** Demographics and dispositional mindfulness facets predicting mental help-seeking attitudes.

		B	SE B	β	t
**Model 1 (Demographics): *R =* 0.12, *R*** **^2^ = 0.01, *F* for change in *R*** **^2^ = 0.85**					
	Constant	43.09	3.02		14.28
	Age	0.07	0.06	0.07	1.24
	Women ref	1.25	1.36	0.06	0.92
	Married	0.07	1.52	0.00	0.05
	Education	0.27	0.31	0.06	0.86
	Yearly Income	0.08	0.53	0.01	0.16
**Model 2 (Demographics and Predicting Factors): *R* = 0.30, *R*** **^2^ = 0.09, Δ*R*** **^2^ = 0.07, *F* for change in *R*** ^2^ = 4.67 **					
	Constant	28.18	4.64		6.07
	Age	0.00	0.06	0.00	0.03
	Women ref	1.75	1.35	0.08	1.29
	Married	0.66	1.50	0.03	0.44
	Education	0.44	0.31	0.09	1.44
	Yearly Income	−0.21	0.52	−0.03	−0.41
	OB	0.20	0.22	0.06	0.90
	DS	0.07	0.19	0.03	0.38
	AA	0.52	0.18	0.21 **	2.92
	NJ	−0.03	0.17	−0.01	−0.15
	NR	0.27	0.16	0.10	1.63

*Note:* OB = Observe facet; DS = Describe facet; AA = Act with Awareness facet; NJ = Non-Judgment facet; NR = Non-Reactivity facet. All values have been rounded up to one decimal place. ** = significant at the 0.01 level.

**Table 8:** Moderating effects of the five FFMQ facets on the relationship between mental health literacy and help-seeking attitudes.

Facet (W/Moderator)	R^2^ (Model)	b (Interaction)	SE (Interaction	*p* (Interaction)	ΔR^2^ (Interaction)	X→Y at Low W (b, *p*)	X→Y at Mean W (b, *p*)	X→Y at High W (b, *p*)
OB	0.15 *	−0.0169	0.0114	0.138	0.0064	Not provided	Not provided	Not provided
DS	0.16 *	0.0124	0.0093	0.1865	0.005	Not provided	Not provided	Not provided
AA	0.18 *	0.0163	0.0080	0.044	0.0114	0.1766 (*p* = 0.0003)	0.2518 (*p* < 0.0001)	0.3271 (*p* < 0.0001)
NJ	0.15 *	0.0063	0.0079	0.795	0.4273	Not provided	Not provided	Not provided
**NR**	0.19 *	0.0237 *	**0.0237**	0.007 *	**0.0205**	**0.1638 (*p* = 0.0018)**	**0.2684 (*p* < 0.001)**	**0.3730 (*p* < 0.001)**

*Note:* OB = Observe facet; DS = Describe facet; AA = Act with Awareness facet; NJ = Non-Judgment facet; NR = Non-Reactivity facet. All values have been rounded up to one decimal place. * = significant at the 0.05 level. All values are rounded up to one decimal place. The critical alpha for analysis was 0.01. Bold lines indicate significant interaction below the adjusted critical alpha. Interaction b = unstandardized coefficient. Δ*R^2^* indicates the additional variance explained by the interaction term. Conditional effects are presented for low (M − 1 SD), mean, and high (M + 1 SD) values when the interaction is significant.

**Table 9:** Mediating effects of general self-efficacy in the relationship between five facets of FFMQ and help-seeking attitudes.

Facet (Predictor)	a (X→M)	b (M→Y)	c (Total)	c′ (Total)	Indirect Effect	99% CI (Indirect)	*p* (Mediation Sig at 0.01)
**OB**	0.4144 **	0.3605 **	0.4421 (*p* = 0.03) *	**0.2927 (*p* = 0.16)**	**0.1494**	**[0.0033, 0.3848]**	**Yes**
DS	0.6320 **	0.2862	0.4692 **	0.2884	0.1809	[−0.0542, 0.4634]	No
AA	0.3622 **	0.2856	0.5715 **	0.4680 **	0.1034	[−0.0121, 0.2665]	No
**NJ**	0.2840 **	0.3530 **	0.3270 (*p* = 0.0168) *	**0.2268 (*p* = 0.1022)**	**0.1002**	**[0.0088, 0.2305]**	**Yes**
NR	0.7211 **	0.3170	0.4165 **	0.1879	0.2286	[–0.0486, 0.4990]	No

*Note:* OB = Observe facet; DS = Describe facet; AA = Act with Awareness facet; NJ = Non-Judgment facet; NR = Non-Reactivity facet. All values have been rounded up to one decimal place. Critical alpha for analysis is 0.01. Bold lines indicate significant indirect (mediation) effect below adjusted critical alpha. ** significant at 0.001 level, * significant at 0.05 level.

## Data Availability

Data are contained within the article.
